# High-resolution genotyping of *Pseudomonas aeruginosa *strains linked to acute post cataract surgery endophthalmitis outbreaks in India

**DOI:** 10.1186/1476-0711-4-19

**Published:** 2005-12-12

**Authors:** Prashanth Kenchappa, Virender S Sangwan, Niyaz Ahmed, K Rajender Rao, Avinash Pathengay, Annie Mathai, Tarannum Mansoori, Taraprasad Das, Seyed E Hasnain, Savitri Sharma

**Affiliations:** 1 Pathogen Evolution Group, Centre for DNA Fingerprinting and Diagnostics (CDFD), Nacharam, Hyderabad, India; 2 L V Prasad Eye Institute (LVPEI), Banjara Hills, Hyderabad, India; 3 Laboratory of Molecular and Cell Biology, Centre for DNA Fingerprinting and Diagnostics (CDFD), Nacharam, Hyderabad, India; 4 Jawaharlal Nehru Centre for Advanced Scientific Research (JNCASR), Jakkur, Bangalore, India

## Abstract

**Background:**

Investigation of two independent outbreaks of post cataract surgery endophthalmitis identified the reservoir of epidemic strains of *P*. *aeruginosa*.

**Methods:**

Patient isolates cultured from vitreous fluid of all the nine cases and from the peripheral devices of phacoemulsification machine were subjected to high-resolution Fluorescent Amplified Fragment Length Polymorphism (FAFLP) analysis.

**Results:**

FAFLP based genotyping of the isolates confirmed nosocomial transmission. Although biochemical characterization and antibiotic susceptibility profiles grouped all the isolates together, FAFLP based genotyping revealed that, all the outbreak isolates were derived from 2 different strains, with independent origins. One group of isolates was traced to phacoprobe and the second one to the internal tubing system of the phacoemulsification machine used in cataract surgery. *In silico *analysis indicated possible evolution in both the clusters of *P. aeruginosa *isolates due to genetic polymorphisms. The polymorphisms were mapped to gene products (cell envelope, outer membrane proteins) possibly having significant role in pathogenesis.

**Conclusion:**

The present study is probably the first one to apply FAFLP typing successfully to investigate outbreaks of postoperative endophthalmitis (POE) in an ophthalmic setting, which was able to identify the source, and helped to make rational decisions on sterilization procedures that halted more cases of infection in these hospitals.

## Background

Cataract extraction is one of the commonest surgical procedures performed on large number of patients worldwide. Post cataract surgery endophthalmitis is a serious sight threatening complication and no effort to prevent it can be too intense. Over the years, the incidence has reduced to 0.06%, largely owing to the advances made in sterilization procedures and understanding of the modes of post surgical infections [1]. The sources of infection leading to endophthalmitis have been traced to conjunctival flora of the patients, contaminated irrigation fluids, intraocular lenses and phacoemulsifiers [1].

Tools of source detection in an outbreak of postoperative endophthalmitis (POE) have moved much beyond culture of suspected samples and antibiotic sensitivity testing of the underlying organisms. Conclusive correlation of suspected organism(s) and the source are necessary to obtain useful information for outbreak investigation. A variety of techniques have been used to investigate the outbreaks including ribotyping and pulsed-field gel electrophoresis (PFGE) [2]. We report herein the evaluation of two outbreaks of POE that were investigated for detection of the source of infection using fluorescent amplified fragment length polymorphism (FAFLP). Apart from demonstrating the utility of a FAFLP technique in outbreak investigations, this report also seeks to emphasize the threat of POE resulting from break in sterility during phacoemulsification procedures, even under best of circumstances.

## Methods

At the time of first outbreak, on June 8, 2003, the Infection Control Committee (ICC) at the LV Prasad Eye Institute (LVPEI), Hyderabad, India was alerted about a possibility of outbreak of POE in 4 of 14 patients undergoing cataract surgery. Only 2 of these 4 patients underwent vitrectomy for the management of POE. Table 1 describes the clinical characteristics of these 2 patients. Preliminary data were obtained telephonically from the surgeon at the satellite hospital and members of ICC visited the hospital for investigation. During the second outbreak, 7 of 15 patients undergoing cataract extraction on July 14, 2003 at LVPEI were suspected to have developed POE and the ICC was notified immediately (Table 1). The ICC members inspected the operating room, sterilization room, preoperative and postoperative areas. Vitreous aspirates/biopsy were collected from all patients and processed as described earlier [3]. Samples were collected from suspected materials/machines that could be the likely source.

**Table 1:** Clinical characteristics of the patients who had cataract extractions from two independent outbreaks caused by *P*. *aeruginosa*. All patients underwent phacoemulsification with intraocular lens (IOL) implantation.

Patient No.	Sex/Age	Systemic disease	Clinical specimen	Date of isolation	Designated strain number	Outcome BCVA ^a ^and comment
*Outbreak 1*						
1	70/F	Nil	Vitreous	04-6-04	L-1130/03	6/120, poor
2	48/F	Nil	Vitreous	05-06-04	L-1147/03	No PL^b ^poor
*Outbreak 2*						
1	55/M	DM, HT ^c^	Vitreous	16-07-04	L-1443/03	6/9 good
2	69/M	Nil	Vitreous	16-07-04	L-1445/03	6/15 good
3	75/M	Nil	Vitreous	16-07-04	L-1446/03	No PL poor
4	70/M	HT	Vitreous	16-07-04	L-1447/03	6/6 good
5	62/M	Nil	Vitreous	16-07-04	L-1449/03	6/12 good
6	62/F	Nil	Vitreous	16-07-04	L-1450/03	No PL poor
7	60/M	HT	Vitreous	16-07-04	L-1455/03	6/6 good

^a ^BCVA-Best corrected visual acuity;^b ^PL – Perception of light; ^c ^DM – Diabetes mellitus, HT-Hypertension

### Surveillance samples

A total of 23 samples were collected from the operating rooms at both sites of outbreaks. In the first outbreak a total of six samples from the phacomachine, five samples from various parts of the operating room and water samples were processed. In particular, samples were swabs from phacoprobe, internal tubings of phacomachine, and Ringer's lactate irrigation solution. In addition, testing of the autoclaves using biological indicators was also performed. The sampling process was repeated and checked for growth after thorough servicing of the instruments and terminal sterilization of the operating room.

Similarly, 12 samples were collected in the second outbreak from phacomachines and different parts of the operating room. In particular, samples were swabs from phacoprobe, internal tubings of phacomachine, viscoelastic material and Ringer's lactate irrigation solution. All four steam sterilizers and two ethylene oxide sterilizers were tested using biological indicators. The sampling process was repeated as in the first outbreak.

All the positive cultures were subjected to biochemical testing for identification and characterization. Preliminary identification was as per the standard microbiological procedures. Automated Biotyping API 20NE (bioMerieux Inc., USA) was used for phenotypic identification. Antibiotic susceptibility testing was performed by using Kirby-Bauer disc diffusion method wherein the following antibiotics were tested: amikacin, ceftazidime, cefazolin, chloramphenicol, ciprofloxacin and gentamicin. Results of antibiotic susceptibility were interpreted according to the NCCLS guidelines [4]**.**

All the bacterial isolates were subjected to FAFLP. Genomic DNA isolation and FAFLP analysis were based on the AFLP and FAFLP methods described earlier [5-7]. The AFLP technique is based on the selective PCR amplification of restriction fragments from a total digest of genomic DNA [7]. The technique involves 3 steps: (i) restriction of the DNA and ligation of oligonucleotide adapters, (ii) preselective amplification of sets of restriction fragments and (iii) selective amplification, wherein additional selective nucleotides were added to the preselective PCR primers that will reduce the total number of bands by four fold with each additional selective base. Furthermore, this addition always results in a fingerprint, which was a subset of the original fingerprints and it is easy to analyze them due to less number of bands. Thus, AFLP uses PCR to selectively amplify defined subsets of DNA restriction fragments from across the whole genome. In its fluorescent form (FAFLP), one of the selective PCR primers are fluorophore labeled, making the amplified fragments visible to an automated DNA sequencer [8]**.**

In brief, genomic DNA digestions were performed using restriction enzymes *EcoR*I and *Mse*I. The sequences of the *EcoR*I adapters were 5' CTCGTAGACTGCGTACC 3' and 3' CATCTGACGCATGGTTAA 5', while those of the *Mse*I, adapters were 5' GACGATGAGTCCTGAG 3' and 3' TACTCAGGACTCAT 5' [7]. For FAFLP secondary selective PCR, forward primer for the *Mse*I adapter site contained a selective nucleotide base C and the nonselective reverse primer for the *EcoR*I adapter site was labeled with a fluorophore (*EcoR*I+0 and *Mse*I+C). These primers were obtained from commercial source (AFLP Microbial Fingerprinting kit; California). FAFLP electropherograms were analyzed using Genescan™ 3.7 and Genotyper™ 3.7 software packages (PE Biosystems) as described earlier [6]. Genescan electropherograms of all the isolates were visually analyzed by superimposing color-coded amplitypes of isolates and different FAFLP profiles were identified based on the presence or absence of monomorphic and polymorphic bands. The percentage similarities/differences between FAFLP patterns were calculated using the Dice correlation coefficient. Cluster analysis was performed using the unweighted pair group method with arithmetic averages (UPGMA) algorithm [9].

## Results

Vitreous samples of all 9 patients yielded significant growth of *P*. *aeruginosa*. *P. aeruginosa *was also isolated from the phacoprobe and internal tubings of phacoemulsification machine used during surgeries in the first and second outbreak, respectively. Rest of the environmental samples were sterile. Isolates belonging to the first outbreak produced green pigment along with metallic sheen, whereas isolates from second episode were more mucoid in nature devoid of characteristic blue-green pigmentation of *P. aeruginosa *species. Antimicrobial susceptibility profiles of all the isolates obtained from both the outbreaks showed identical profiles wherein they were resistant to cefazolin and sensitive to amikacin, ceftazidime, chloramphenicol, ciprofloxacin and gentamicin.

**Figure 1:**
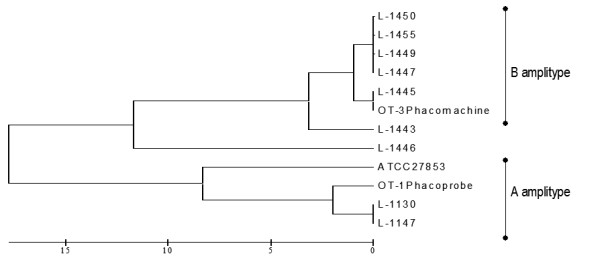
UPGMA tree showing similarity levels deduced from the genotyper data derived from FAFLP profiles showing all the 11 strains isolated during two outbreaks along with the reference ATCC strain of *P*. *aeruginosa*. The scale at the bottom of the figure indicates genetic distance between the isolates.

**Figure 2:**
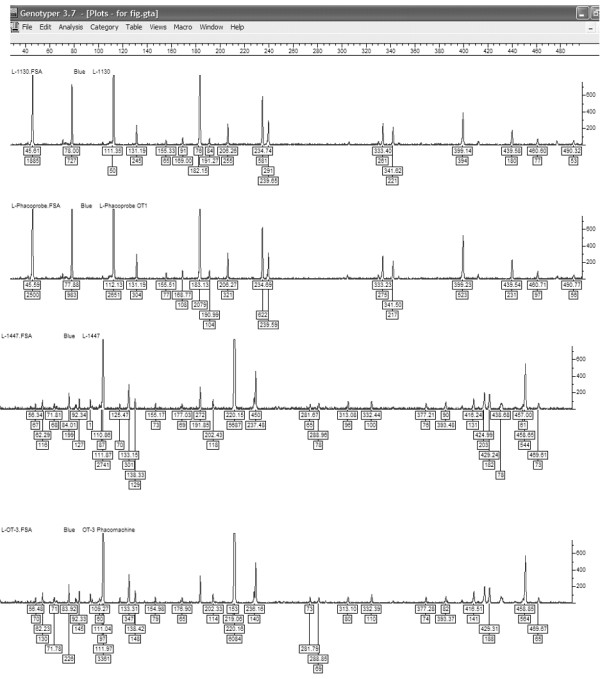
*P. aeruginosa *strain specific FAFLP profiles for *Mse*I+C selectivity tested, showing number and fragment sizes as well as the peak heights. The genotyper plots of representative clones of FAFLP amplitypes A *&*B of the two outbreaks. FAFLP patterns in order (top to bottom) are L-1130 (Amplitype A), L-OT1 phacoprobe (Amplitype A), L-1447 (Amplitype B), and L-OT3 Phacomachine (Amplitype B).

Genescan™ electropherograms of all the isolates were visually analyzed for detection of presence or absence of monomorphic and polymorphic bands. The single primer combination used (*EcoR*I+0 and *Mse*I+C) for FAFLP, generated a total of 19 to 36 differently sized fragments experimentally ranging in size from 50 to 500 bp from all the isolates. Forty-nine monomorphic and 12 polymorphic fragments were generated from a total of 11 strains analyzed. Cluster analysis generated 2 distinct clusters among the strains having discrete genetic lineages and they were designated as amplitype A and amplitype B (Figure 1). Isolates belonged to amplitype A, which represented the cause of first outbreak, generated 19 to 21 fragments. Twenty-one fragments were monomorphic and 6 bands were polymorphic in this group. Isolates belonging to amplitype B that represented the cause of second outbreak generated a total of 26 to 32 fragments, of which 28 were monomorphic and only 8 bands were polymorphic. Representative clonal specific FAFLP amplitypes are depicted in figure 2. Distribution of fragments among different isolates within each cluster (type strain) did not vary significantly.

Under all experimental conditions, the characteristic FAFLP profiles of the strains were reproducibly generated reassuring their consistency over time. FAFLP performed for replicates of DNA derived before and after five subcultures of the standard ATCC strain were examined. They shared a minimum of 98% inter-gel similarity for approximately 35 fragments. Therefore, individual isolates of *P. aeruginosa *that shared ≥ 98% similarity (having ≤ 2% of difference) are likely to be identical clones [10]. Unlike the similarity in antibiotic profile, all the 11 isolates from the 2 outbreaks occurring at two separate hospitals formed two distinct genetic clusters having 18% of genetic divergence between them, when evaluated by cluster analysis (Figure 1). Two isolates that were recovered from the first episode were 100% identical between themselves and 98% identical to a third isolate linked to the phacoprobe. Majority of isolates (7 of 8) of amplitype B had less than 3% of genetic difference. These 7 isolates obtained from the second outbreak were closely related, appeared clonal in origin (Figure 1). Six isolates within this cluster were 99% identical representing identical clones. Further, within these 6 isolates, 4 were 100% identical.

**Figure 3:**
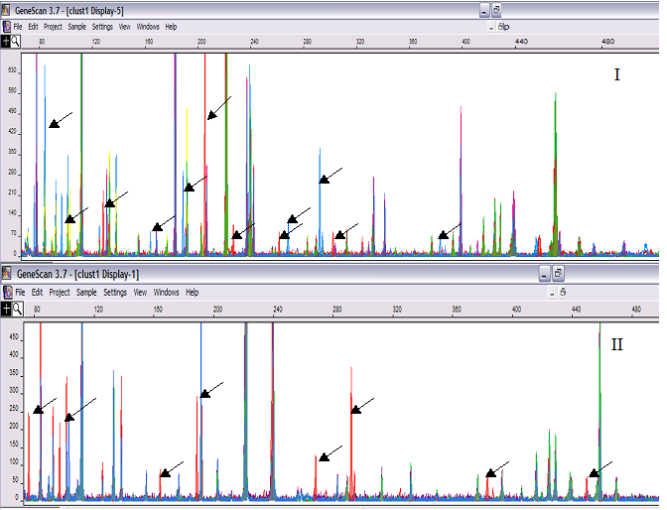
**I **Genescan-derived FAFLP profiles from isolates belonged to two outbreaks. FAFLP profiles with similar gel mobility conditions with equal data points were color-coded and superimposed to visualize differentially amplified fragments (visible as peaks; peak height indicates the quantity of amplicon generated, and peak position indicates size in base pairs). Polymorphic fragments are marked with the arrows. The horizontal scale indicates size in bp, while the vertical scale indicates level of fluorescence incorporated (peak intensity). **II **strains from amplitype B – Polymorphic fragments in base pairs – 76, 96, 152, 188, 256, 292, 383 and 445.

Predictive *in silico *methods used on genome sequence of *P. aeruginosa *PA01 generated a total of 51 fragments of sizes between 50 to 500 bp upon selective PCR with single selectivity of *Mse*I +C [11]. Comparative *in silico *analysis with predictive annotation of sequenced PA01 strain was performed wherein isolates belonged to the two independent outbreaks were subjected to FAFLP, and the results were extrapolated to the computer-predicted AFLP data of the *P. aeruginosa *PA01 sequence. Differential amplification of 6 to 12 genomic regions (Figure 3–I) among the isolates and their extrapolations revealed genetic differences between the isolates. While comparing GeneScan profiles of isolates belonging to two outbreaks, L1446, L1443 and ATCC strain showed amplification of 12 bands that were absent in rest of the isolates. The corresponding polymorphisms were mapped to genes coding for probable porin gene (PA4137), probable ATP-binding component of ABC transporter (PA4909), rplA gene coding for 50S ribosomal protein (PA4273) and many conserved hypothetical protein ORFs namely PA0234, PA1370, PA1788, PA2228, PA3350, PA1091, and PA4203. When comparison of isolates belonging to amplitype B alone was performed, eight polymorphic bands were observed in isolates L1446 and L1443 (Figure 3–II). All the polymorphisms observed were reproducible.

## Discussion

Despite low incidence, POE remains a serious complication [1]. All patients included in this study required pars plana vitrectomy with intraocular antibiotics and corticosteroid injection. One patient required therapeutic penetrating keratoplasty with intraocular lens explantation after nine days of treatment. While intraocular amikacin and vancomycin with dexamethasone were administered at the time of presentation in all patients, two patients required repeat injections of ceftazidime and dexamethasone. All patients were given ciprofloxacin eye drops topically along with mydriatics and prednisolone acetate eye drops. Six patients were also treated with oral ciprofloxacin and corticosteroid. The outcome was satisfactory in five of our patients and it was poor in remaining four patients.

Outbreaks of *P. aeruginosa *POE most likely have an exogenous origin, as they are not normal commensal on skin and conjunctiva [1]. Understanding the relative importance of the routes of colonization is crucial for the development of effective preventive remedies against *P. aeruginosa *POE. The source of infection and routes of contamination are important issues in any hospital [2]. In the present study, a reservoir source was suspected in each of these independent outbreaks. Following a thorough sampling of operating room environment and equipment, *P. aeruginosa *were isolated from phacoprobe at the first hospital and from the internal tubing of phacoemulsification surgical equipment in the second hospital. All the isolates obtained from the clinical and environmental sources from both the outbreaks showed identical antibiotic susceptibility patterns, implying a single source for both the outbreaks, which was most unlikely given the distance in time of occurrence and separate physical location of the hospitals. On the other hand, FAFLP analysis clearly showed that there were two independent sources involved. Molecular typing methods are necessary for confirming the source of an outbreak [1]. In the present study, FAFLP conclusively identified the isolate from surgery equipment to be the source for infection in both the outbreaks. Nosocomial contamination and acquisition was proven in this study. Clustering of the isolates was close enough to explain their clonal expansion in the respective hospital settings. Distinct identity of the two strain types involved in the outbreaks was obvious with 18% genetic difference between them. This was highly significant with additional distinctive phenotypic characteristics like green metallic sheen and mucoid colonies, notwithstanding the similarity in antibiotic susceptibility.

DNA typing methods have emerged as more practical and reliable option for the investigation of outbreaks. AFLP analysis has been reported to provide sufficient discriminatory power comparable to the PFGE for the investigation of *P. aeruginosa *outbreaks [5,6]. Our study provides additional proof for the above observations. Minor variations are more frequent in *P. aeruginosa *isolates with abundant mutations and recombination that contemplates wide spectrum of microevolution in a shorter duration [5,6]. These variations were more evident in our study wherein only 6 to 8 fragment difference was observed within the isolates of different amplitypes. Distribution of fragments among different isolates within each cluster (Amplitype A & B) did not vary significantly.

Comparative *in silico *analysis with predictive annotation of sequenced PA01 strain showed lack of amplification of the 6 to 12 genomic regions in many isolates belonging to both the clusters (L1445, L1447, L1449, L1450, L1455, OT3, L1130, L1147 and OT1) and this might be certainly due to the mutation (indels or point mutation) in the DNA region bearing *EcoRI *and *MseI *restriction sites. Genetic polymorphisms mapped to genes coding for probable porin gene (PA4137), probable ATP-binding component of ABC transporter (PA4909) and COG functional prediction of ORFs PA1091 and PA3350 as glycerophosphate transferase involved in teichoic acid biosynthesis and flagellar based body P-ring biosynthesis protein respectively, appears to be significant as all these four gene products are localized in cell envelope and outer membrane of the organism [12]. In addition, COG prediction of ORF PA4203 as probable lysR transcriptional regulator, is known to be very similar to the periplasmic binding proteins. Modification of these genes in above-mentioned isolates might possibly indicate enhanced ability of these isolates to cause POE through alterations of their outer membrane porin and efflux proteins [13].

Genetic polymorphisms mapped to ORFs PA4137, PA4909 and PA2228 seem to be very important. PA4137 is 52% similar to the oprE gene product of *P. aeruginosa *that is homologous to OprD, which is known to be involved in permeability of imipenem and basic amino acids [14,15]. PA4909 is 74% homologous to braG gene product of *P*. *aeruginosa*, which is cell membrane protein bearing ATP- binding domains [16]. COG prediction of PA2228 as AmpC, beta-lactamase class C family of protein is usually associated with beta lactam resistance in *P. aeruginosa *[17]. Lack of amplification of these three regions in the closely related clones of amplitype A and B appears to be due to mutation under different stress conditions prevailing in the host. Further studies are clearly needed to ascertain functional role of these polymorphisms in ocular infection.

## Conclusion

In order to prevent nosocomial outbreaks, apart from constant surveillance of the hospital environment and stringent infection control measures, it is important to apply high utility programs such as FAFLP to determine source of infection. This study used FAFLP typing to identify the source of infection in two outbreaks of POE. In addition, FAFLP data extrapolated *in silico*, would help in detecting genetic variations among the isolates

## Abbreviations

FAFLP: Fluorescence amplified fragment length polymorphisms

POE: Postoperative endophthalmitis

PFGE: Pulse Field Gel Electrophoresis

ICC: Infection Control Committee

UPGMA: unweighted pair group method with arithmetic averages

COG: Clusters of Orthologous Groups

## Competing interests

The author(s) declare that they have no competing interests

## Authors' contributions

**PK **– Designing the study, performed FAFLP for the outbreak isolates. Analysis of FAFLP data, phylogenetic analysis and drafting the paper

**VSS **– Clinician who performed cataract surgeries, provided clinical data and evaluated the manuscript.

**NA **– Supervised the study, provided laboratory support and edited the manuscript.

**KRR **– Assisted in performing FAFLP and FAFLP analysis.

**AP **– Examined and treated the affected patients.

**AM **– Examined and treated the patients and also accompanied SS for investigations of *Outbreak 1*. One of the members of the ICC.

**TM **– Performed cataract surgeries using phacoemulsification.

**TD **– Examined and treated the affected patients as well as manuscript evaluation.

**SEH **– Critical analysis of manuscript, editing and final presentation.

**SS **– Co-designing the study, identification of outbreaks isolates and their distinct phenotypic characters, antimicrobial susceptibility testing, assisted in writing the paper. One of the members of the ICC

All authors read and approved the final manuscript.
